# Double primary bronchogenic carcinoma of the lung and papillary thyroid carcinoma: a case report

**DOI:** 10.1186/1752-1947-2-309

**Published:** 2008-09-23

**Authors:** Jen-Hsun Cheng, Ying-Chieh Huang, Chih Kuo, Yih-Shyong Lai, Tzu-Ching Wu, Thomas Chang-Yao Tsao, Shi-Ping Luh, Chong-Bin Tsai

**Affiliations:** 1 Department of Thoracic Medicine and Surgery, Chia-Yi Christian Hospital, Chia-Yi 600, Taiwan; 2 National Chung-Cheng University, Min-Hsiung, Chia-Yi 621, Taiwan; 3 Department of Pathology, Chung-Shan Medical University Hospital, Taichung City, Taiwan; 4 Department of Medicine, Chung-Shan Medical University Hospital, Taichung City, Taiwan

## Abstract

**Introduction:**

Double primary bronchogenic carcinoma and papillary carcinoma of the thyroid are extremely rare. We describe the case of a patient who underwent surgical resection for these two cancers.

**Case presentation:**

A 56-year-old man presented to our hospital complaining of a cough with blood-tinged sputum. A slowly growing mass in the left lobe of the lung had been noted for about 1 year. He underwent video-assisted thoracic surgery of the left lower lobe and mediastinal lymph node dissection through an 8 cm utility incision. Pathology revealed a well-differentiated adenocarcinoma and the dissected lymph nodes were negative for malignancy. He also complained of a mass in his neck, which had grown slowly for over 5 years. A computed tomography scan of the neck revealed a left thyroid mass compressing the trachea towards the right side. There was no cervical lymphadenopathy. A left thyroid lobectomy was performed and pathology revealed a papillary carcinoma. Thus, he underwent a second operation to remove the right lobe of the thyroid. He underwent subsequent adjuvant chemotherapy.

**Conclusion:**

In a review of the literature, it appears that there has only been one previously reported case of these two cancers, which was in Japan. The relationship between these two cancers is still unclear, and more case reports are required to determine this relationship.

## Introduction

The incidence of multiple primary malignancies has increased in recent years [1]. Commonly occurring malignancies accompanying primary lung cancer are found in the lung, upper respiratory tract, breast, esophagus, colon, rectum, stomach and cervix [2]. Double primary thyroid and lung cancers have rarely been reported [3-5]. Here we describe a case of a patient with double primary lung and thyroid cancers who underwent curative surgical resection.

## Case presentation

**Figure 1:**
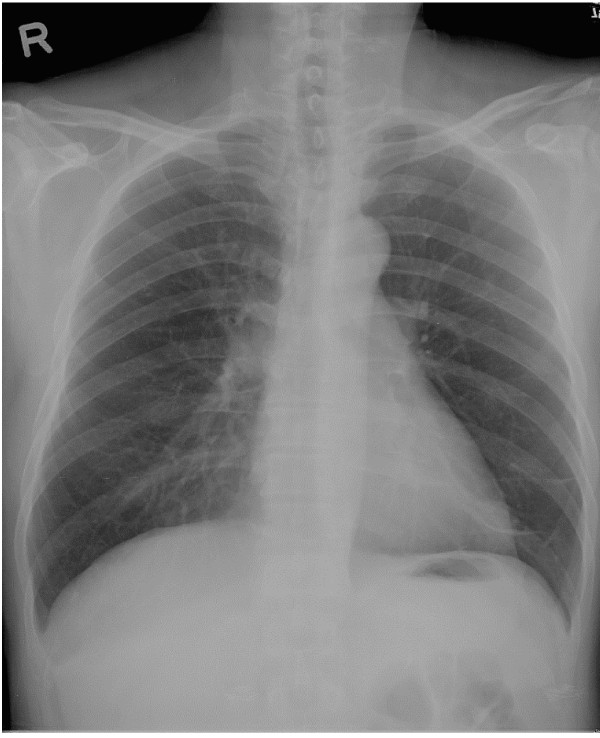
Chest X-ray showing a mass shadow over the left lower lung field (arrow).

**Figure 2:**
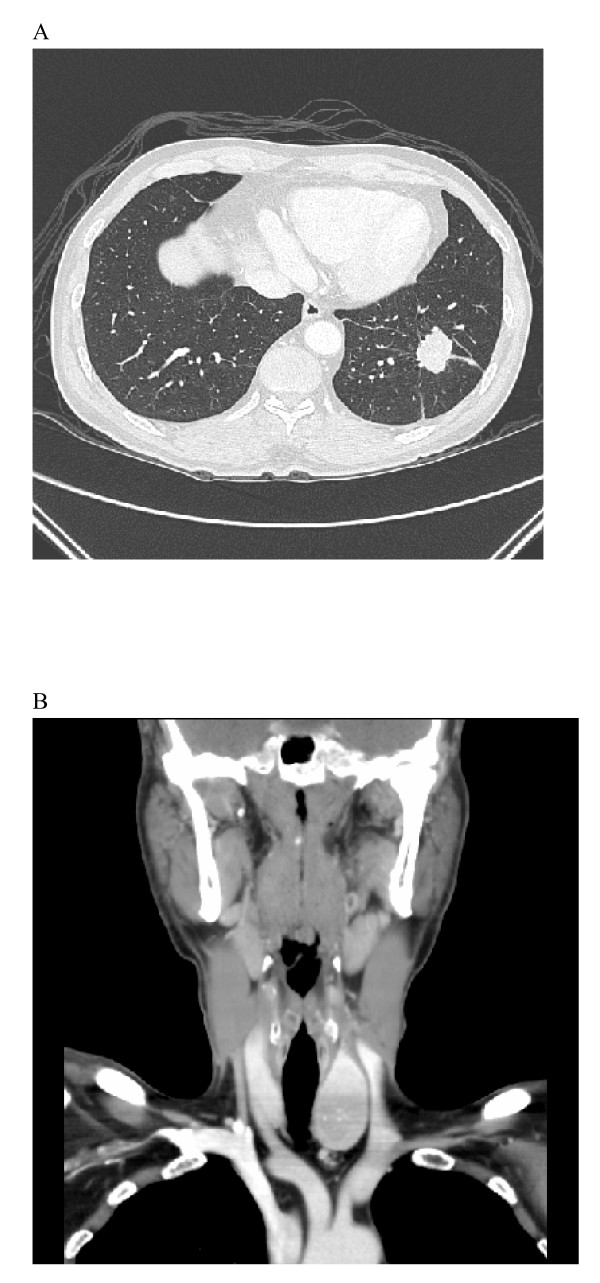
Computed tomography (CT) showing a nodule, 3.5 cm in diameter, within the left lower lobe of the lung with pleural retraction (A), and a mass 5 cm in diameter within the left lobe of the thyroid (B).

A 56-year-old man, who was well except for hypertension of over 10 years duration for which he received regular treatment, presented to our hospital complaining of intermittent chest tightness for a month. The chest tightness, which had been aggravated in the previous week, was located in the left precordal area, and was persistent in character and induced by exercise. On examination, the patient was slightly anxious but generally well. A mass was noted over the left side of his neck and he stated that this had been present for more than 4 years. He did not pay attention to it initially because it had been growing very slowly. However, he had noted mild labor on respiration in recent months. No abnormal breath sounds or heart murmurs were noted. The hemogram and blood chemistry were normal. Chest X-ray revealed a mass in the left lower lung field (Figure 1). Computed tomography (CT) revealed a nodule, 3.5 cm in diameter, in the left lower lobe of the lung with pleural retraction (Figure 2A), and also a mass, 5 cm in diameter, within the left lobe of the thyroid (Figure 2B). Fiberoptic bronchoscopy was negative for any intraluminal lesions. An adenocarcinoma of the lung was confirmed by CT-guided biopsy. A whole body bone scan was negative for skeletal metastasis. A fluorodeoxyglucose-positron emission tomography revealed a hypermetabolic focus in the left lower lobe of the lung and in the left lobe of the thyroid. He was admitted for further evaluation and treatment.

The patient underwent a left lower lobectomy to remove the pulmonary mass and mediastinal lymph node dissection through video-assisted thoracic surgery with a minithoracotomy. The resected specimen revealed a 3.5 × 3 × 2.5 cm elastic-firm, high-cellular mass with pleural retraction. All of the nine dissected mediastinal lymph nodes were negative. Grossly, the localized lung tumor was a well-differentiated adenocarcinoma which was shown pushed against the pleura with lymphocytic infiltration, but not penetrating it. Microscopically, the tumor was arranged in a glandular structure, composed of neoplastic cells with irregularly enlarged and hyperchromatic nuclei. Some papillary configuration and fused glands were present. The lung adenocarcinoma revealed on immunohistochemistry surfactant apoprotein A positivity for tumor cells as well as normal alveolar cells. The patient's postoperative course was uneventful and he was discharged 9 days after the operation.

**Figure 3:**
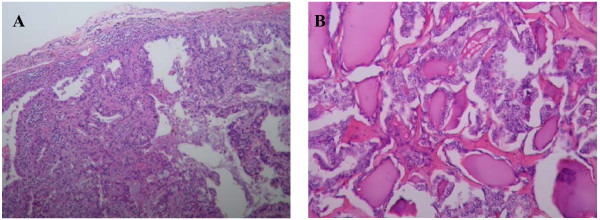
**Well differentiated pulmonary adenocarcinoma. **(A). The tumor is arranged in glandular structure, composed of neoplastic cells with irregularly enlarged and hyperchromatic nuclei. Some papillary configuration and fused glands are present. (H & E stain, 200×). Histopathology of the thyroid tumor reveals papillary structure with ground glass appearance of tumor cell nuclei (B). Some colloid within neoplastic follicles is evident (H & E stain, 200×).

He was later readmitted and underwent a left thyroid lobectomy for what appeared to be a nodular goiter. Microscopy revealed a papillary structure with a ground-glass appearance of tumor cell nuclei. Some colloid within neoplastic follicles was evident. Immunohistochemical staining was positive for tumor cells. The patient underwent a residual radical thyroidectomy. No residual tumor was found in the resected thyroid, parathyroids or cervical lymph nodes. During follow-up, his thyroglobulin level remained low. Hypothyroidism and hypoparathyroidism were noted after the radical thyroidectomy and these symptoms were controlled by thyroid hormone and calcium supplements. The pathology findings confirmed the diagnosis of a double primary pulmonary adenocarcinoma and thyroid papillary carcinoma (see figure 3).

## Discussion

Patients with lung cancer have a high risk of multiple primary malignancies. Other potential sites for multiple primary cancers include the nasopharynx, lungs, large bowel and mammary glands [6]. The incidence of multiple primary malignancies for patients with overall and resected non-small cell lung carcinoma (NSCLC) was 11% and 7–7.4%, respectively [7]. Liu et al. [1] reported that the most common tumors accompanying lung cancer were in the upper aerodigestive tract, followed by colorectal and cervical malignancies. Hsieh et al. [8] reported from the same database that the order of frequency of malignancies for the upper aerodigestive tract was larynx, nasopharynx, esophagus, oral cavity and hypopharynx.

Double primary thyroid and lung carcinomas have been reported only rarely in the literature [3-5]. Shinozaki et al. [9] reported that thyroid carcinoma occurred in 9.7% of patients with multiple primary malignancies, and the most frequent sites for the associated cancers were the breast, uterine cervix and uterine body in women, and the stomach and larynx in men. However, thyroid carcinoma was found with a higher rate of second malignancy (22.7%) than average (4.2%) in autopsy findings, and follicular carcinoma was more frequent among the cancers associated with another tumor (12 out of 20 cases), while in general papillary carcinoma was the most frequent (48 out of 88 cases) [10].

Differential diagnosis for the patient in our case included pulmonary metastasis from the thyroid cancer or vice versa, and both these situations have been reported previously [11]. Pathological iodine-131 uptake will occur in both the primary lung tumor as well as in metastases from the thyroid gland, thus it is not reliable for making a diagnosis [12].

Double primary cancer is the most reasonable diagnosis in our case because there was no evidence of either mediastinal or cervical lymph node metastasis, and the tumors from the two sites had different pathological characteristics.

The associations between these two cancers are still unclear. Mutating proto-oncogenes associated with thyroid carcinoma, such as the ret oncogene, have not been found in patients with lung carcinoma [13]. Furthermore, the environmental factors associated with lung carcinoma, such as smoking or air pollution, have not been not correlated with thyroid carcinoma [14]. Therefore, coincidence is possible in this patient, but further related studies are required to determine where there is an association between these two cancers.

Surgical resection is indicated for either thyroid papillary carcinoma or early to mid stage (before Stage IIIa) non-small cell lung carcinomas (NSCLCs). Therapeutic strategies for the management of double primary thyroid and lung carcinomas, in general, follow their separate guidelines. However, since the progression of a thyroid papillary carcinoma is much slower than that of NSCLCs, in some patients with limited survival removal of the thyroid neoplasm may not be considered appropriate [4]. In the patient described in this case report, since there were no lymph nodes involved or distant metastasis, surgical resection of both lesions was the therapy of choice.

## Conclusion

A patient with a double primary thyroid papillary carcinoma and pulmonary adenocarcinoma was successfully treated by surgical resection of both tumours. Reports of related cases in the previous literature are rare.

## Abbreviations

CT: computed tomography; NSCLC: non-small cell lung carcinoma

## Competing interests

The authors declare that they have no competing interests.

## Authors' contributions

S-PL was the attending doctor, carried out the surgical procedure and literature review and wrote the manuscript. CK and Y-SL performed the pathological examination and assisted in writing the report. T-CW and TC-YT were the chest physicians providing care to this patient. Y-CH and C-BT revised and provided comments on the manuscript. J-HC collected the data and literature review, and wrote the manuscript.

## Consent

Written informed consent was obtained from the patient for publication of this case report and any accompanying images. A copy of the written consent is available for review by the Editor-in-Chief of this journal.
